# Propofol inhibits parthanatos via ROS–ER–calcium–mitochondria signal pathway in vivo and vitro

**DOI:** 10.1038/s41419-018-0996-9

**Published:** 2018-09-17

**Authors:** Hanhui Zhong, Rui Song, Qiongni Pang, Yawei Liu, Jinling Zhuang, Yeming Chen, Jijie Hu, Jian Hu, Youtan Liu, Zhifeng Liu, Jing Tang

**Affiliations:** 1 The Department of Anesthesia, Affiliated hospital of Guangdong Medical University, Zhanjiang, Guangdong China; 2 The Department of Anesthesia, Nanfang Hospital, Southern Medical University, Guangzhou, Guangdong China; 3 Center for Perinatal Biology, Division of Pharmacology, Department of Basic Sciences, Loma Linda University School of Medicine, Loma Linda, CA USA; 4 Laboratory for Precision Neurosurgery, Nanfang Hospital, Southern Medical University, Guangzhou, Guangdong China; 5 The Department of Anesthesia, The Third Affiliated Hospital, Southern Medical University, Guangzhou, Guangdong China; 6 The Department of Orthopaedics and Traumatology, Nanfang Hospital, Southern Medical University, Guangzhou, Guangdong China; 7 Heart, Lung, Blood, and Vascular Medicine Institute, University of Pittsburgh, 200 Lothrop St, Pittsburgh, PA 15216 USA; 8 The Department of Anesthesiology, Shenzhen Hospital, Southern Medical University, Shenzhen, Guangdong China; 9 The Department of Critical Care Medicine, General Hospital of Guangzhou Military Command, Guangzhou, China; 10 Guangdong Provincial Key Laboratory of Molecular Oncologic Pathology, Southern Medical University, Guangzhou, Guangdong China

## Abstract

Parthanatos is a new form of programmed cell death. It has been recognized to be critical in cerebral ischemia–reperfusion injury, and reactive oxygen species (ROS) can induce parthanatos. Recent studies found that propofol, a widely used intravenous anesthetic agent, has an inhibitory effect on ROS and has neuroprotective in many neurological diseases. However, the functional roles and mechanisms of propofol in parthanatos remain unclear. Here, we discovered that the ROS–ER–calcium–mitochondria signal pathway mediated parthanatos and the significance of propofol in parthanatos. Next, we found that ROS overproduction would cause endoplasmic reticulum (ER) calcium release, leading to mitochondria depolarization with the loss of mitochondrial membrane potential. Mitochondria depolarization caused mitochondria to release more ROS, which, in turn, contributed to parthanatos. Also, we found that propofol inhibited parthanatos through impeding ROS overproduction, calcium release from ER, and mitochondrial depolarization in parthanatos. Importantly, our results indicated that propofol protected cerebral ischemia–reperfusion via parthanatos suppression, amelioration of mitochondria, and ER swelling. Our findings provide new insights into the mechanisms of how ER and mitochondria contribute to parthanatos. Furthermore, our studies elucidated that propofol has a vital role in parthanatos prevention in vivo and in vitro, and propofol can be a promising therapeutic approach for nerve injury patients.

## Introduction

Poly (ADP-ribose) (PAR) polymerase-1 (PARP-1) is an essential nuclear enzyme that responds to DNA damage and facilitates DNA repair^1,2^. Once activated, PARP-1 catalyze the transfer of ADP-ribose units from nicotinamide adenine dinucleotide (NAD^+^) to nuclear proteins, including histones and PARP-1 itself, which facilitate DNA repair and cell survival^1–3^. Excessive activation of PARP-1 leads to cellular NAD+ and ATP depletion, results in the accumulation of Poly(ADP-ribose) (PAR) polymers, and causes translocation of apoptosis-inducing factor (AIF) from mitochondria to the nucleus AIF, which is a caspase-independent cell death program, has been designated parthanatos (PARP-1-dependent cell death) to distinguish it from other forms of cell death, such as necrosis, apoptosis, and autophagy^4,5^. Parthanatos has a critical role in many diseases, including ischemia–reperfusion injury in the brain, heart, and other organs; inflammatory injury, reactive oxygen species (ROS)-induced injury, and neurobiological disorders, such as Alzheimer’s disease (AD), Parkinson’s disease (PD), and multiple sclerosis^6–9^.

Excessive activation of PARP-1 is critical in responding to extensive DNA damage due to the attack of genotoxic agents^2^. Some studies demonstrated that ROS contributes to the process of parthanatos caused by genotoxic agents^10–12^. Reduced ROS can inhibit parthanatos in various glioma cells treated with peroxide (H_2_O_2_)^11^. Similarly, inhibition of ROS can suppress parthanatos in embryonic mouse fibroblasts induced by *N*-methyl-*N*′-nitro-*N*′-nitrosoguanidine (MNNG), an alkylating agent that damages DNA^12^. As we all have known, intracellular calcium is an essential messenger in many signaling pathways and has a role in cell death^13^. A study has demonstrated that increasing calcium contributes to PARP-1 activation and neuronal death^14^. However, currently, the two cases of the mechanism of parthanatos remain unknown. First, the origins of ROS and calcium are not adequately demonstrated. Second, the relationship between ROS and calcium is found to be unresolved.

Propofol (2, 6-diisopropylphenol), an intravenous anesthetic agent, is widely used as an anesthetic agent and in intensive care situations. In addition to its sedative effects, propofol has a protective effect against ROS-mediated cell injuries, including apoptosis and autophagy, through inhibiting the production of ROS^15–17^. Also, several studies have shown that it is a neuroprotective agent against cerebral ischemia–reperfusion injury in animal models, which can reduce infarction size and improve neurologic scores^18,19^. Similarly, recent evidence has shown that propofol protects against cerebral ischemia–reperfusion by inhibiting calcium-induced mitochondrial membrane permeability transition and reducing the production of ROS^17,19,20^. Moreover, experimental studies reveal that propofol can inhibit the increase of PARP-1 expression and activity^21,22^. However, the effect of propofol on parthanatos in vivo and in vitro, and the mechanisms involved remain unclear.

Therefore, in this study, we aim to further explore the signaling pathway of parthanatos and the effect of propofol on parthanatos. Here, we found that not only MNNG but also oxygen and glucose deprivation (OGD)/reoxygenation could induce parthanatos. Moreover, we have examined the interactive roles of ROS, endoplasmic reticulum (ER), calcium, and mitochondria in MNNG-induced parthanatos by using molecular inhibitors. We identified that ROS production triggered by MNNG causes ER release of calcium, which leads to mitochondrial depolarization resulting in further release of ROS by the mitochondria. Overproduction of ROS contributes to parthanatos. Furthermore, we found that propofol suppresses parthanatos via inhibiting ROS overproduction, calcium release from ER, and mitochondrial depolarization in vitro (MNNG-induced or OGD/reoxygenation-induced parthanatos) and in vivo (MCAO).

## Results

### MNNG or OGD/reoxygenation induces parthanatos in SH-SY5Y cells or primary neurons

**Fig. 1:**
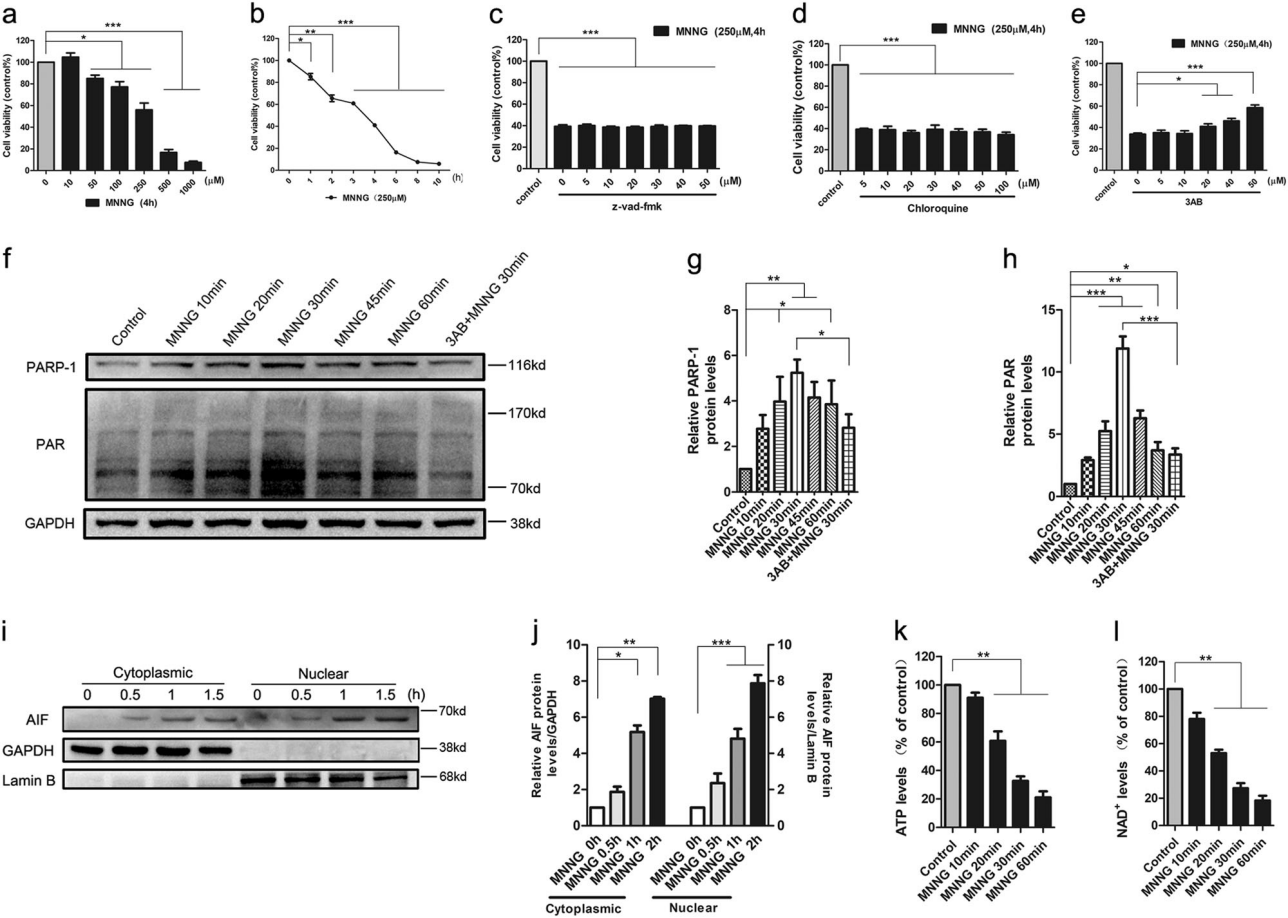
MNNG induces parthanatos in SH-SY5Y cells. SH-SY5Y cells were treated with different concentration of MNNG for 4 h (**a**) or treated with 250 μM MNNG for different time intervals (**b**). SH-SY5Y cells were pre-treated with different concentration of z-vad-fmk (**c**) or chloroquine (**d**) for 1 h before 250 μM MNNG treatment. SH-SY5Y cells were pre-treated with indicated concentrations of 3-AB for 1 h and then exposed to MNNG (250 μM) for 4 h (**e**). The CCK8 assay assessed cell viability. **f** Time course of MNNG-induced PAR accumulation and PARP-1 activation after 250 μM MNNG treatment with or without 3-AB pretreatment. GAPDH was used as a loading control. **g**, **h** Quantification of PARP-1 **g** and PAR **h** expressions. **i** Western blots analysis of AIF amount in both cytoplasm and nuclei of SH-SY5Y cells after 250 μM MNNG treatment for 0.5, 1, and 1.5 h. GAPDH or lamin B was used as loading control separately. **j** Quantification of AIF expression in both cytoplasm and nuclei. The levels of ATP (**k**) and NAD+ (**l**) after treated with 250 μM MNNG for a different time. Each bar represents the mean ± S.E.M based on three independent experiments. *P < 0.05, **P < 0.01, ***P < 0.001

MNNG is thought to be a classical DNA-alkylating agent for inducing parthanatos, and parthanatos has a critical role in the cerebral ischemia–reperfusion injury. However, the mechanism of parthanatos is not well-studied. Here, SH-SY5Y cells were exposed to different concentrations of MNNG for different time intervals to investigate the best time and concentration for MNNG-inducing cell death (Fig. 1a, b). Compared with those in the control group, the average viabilities of SH-SY5Y cells decreased markedly in response to the increase of MNNG doses and the extension of incubating time, indicating that MNNG-induced SH-SY5Y cells death is in a concentration-dependent and time-dependent manner, and 250 μmol/l MNNG had a significant effect on cell death. As shown in Fig. 1c, d, caspase inhibitor z-vad-fmk or autophagy inhibitor chloroquine pretreatment had no effect on cellular viability after MNNG treatment, indicating that MNNG-induced cell death in SH-S5Y5 cells did not include apoptosis or autophagy (Fig. 1c, d).

On the other hand, MNNG increased PAR formation and induced PARP-1 activation, as early as 20 min after MNNG treatment and reached a peak at 30 min (Fig. 1f–h). Moreover, MNNG-induced cell death, PAR formation, and PARP-1 activation could be inhibited by the PARP inhibitor, 3-AB (Fig. 1e–h). As shown in Fig. 1i and j, MNNG also naturally increased the nuclear level of AIF at 1 h after treatment. Besides, MNNG decreased the levels of NAD+ and ATP at 30 min after treatment (Fig. 1k, l). This phenomenon was true for other nerve cells as well. In primary mouse neurons, OGD/reoxygenation induced cell death, which could not inhibit by z-vad-fmk or chloroquine (Figs. 1a). Similarly, in OGD/reoxygenation-induced cell death, the PAR formation, a specific marker of parthanatos, were significantly increased (Figs. 1b, c). All these results indicated that MNNG or OGD/reoxygenation mainly induces parthanatos, but not other forms of cell death.

### ROS contributes to parthanatos

**Fig. 2:**
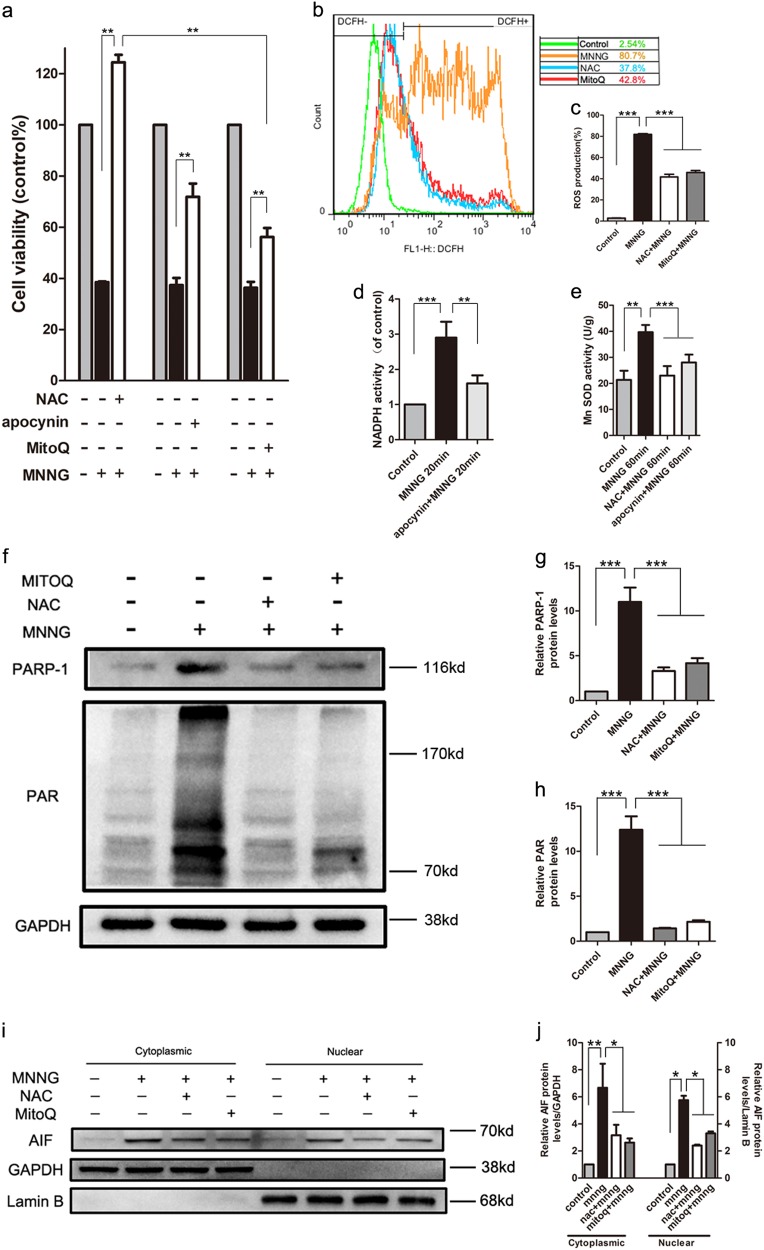
Overproduction of ROS contributes to MNNG-induced parthanatos. **SH-SY5Y cells were treated with MNNG (250 μM) for 4 h with or without 5 mM NAC, 20 nm MitoQ, or 1 μM apocynin pretreatment for 1 h. a** CCK8 assay measured cell viability. **b** FACS analysis of DCFH2-DA fluorescence assessed ROS production. **c** Quantification of ROS production in all groups. **d** ELISA measured NADPH activity. **e** WST-8 assessed Mn-SOD activity. **f** Western blot analyzed PARP-1 activation and PAR generation. GAPDH expression was used as a loading control. **g**, **h** Quantification of PARP-1 (**g**), and PAR (**h**) expressions. **i** Western blot also analyzed AIF amount in both cytoplasm and nuclei. GAPDH or lamin B was used as loading control separately. **j** Quantification of AIF expression in both cytoplasm and nuclei. Each bar represents the mean ± S.E.M. of three individual experiments. *P < 0.05, **P < 0.01, ***P < 0.001

To verify whether ROS has a role in parthanatos, we measured the change of ROS in SH-SY5Y cells after MNNG treatment using redox-sensitive fluorescent probe 29-,79-dichlorofluorescein diacetate (DCFH_2_-DA). As shown in Fig. 5d, ROS was detected as early as 10 min after MNNG treatment and increased markedly in response to the longer time of MNNG treatment, indicating that oxidative stress occurred in MNNG-treated SH-SY5Y cells. Then, cells were pre-treated with antioxidant, NAC, or mitochondria-targeted antioxidant, MitoQ, which degrades intracellular ROS and mitochondria ROS, respectively, before MNNG treatment. We found NAC pretreatment almost entirely reversed the MNNG-induced cell death and ROS outburst (Fig. 2a–c). Moreover, compared with MNNG group, MitoQ pretreatment could also partially inhibit the MNNG-induced cell death and production of ROS (Fig. 2a–c). This phenomenon was true for OGD/reoxygenation-induced parthanatos in primary mouse neurons, NAC and MitoQ could inhibit ROS (Figs. 2a, b). To confirm the origin of ROS, we measure the activity of NADPH and Mn-SOD. We found that MNNG could increase NADPH or Mn-SOD activity since 20 min or up to 60 min, but NAC or apocynin (NADPH oxidases inhibitor) could decrease the activity of NADPH and Mn-SOD (Fig. 2d, e). Besides, apocynin, which inhibit NADPH oxidases, pretreatment could increase the cell viability (Fig. 2a).

To further verify the role of ROS in parthanatos, we observed the effect of ROS on parthanatos-related proteins in MNNG-treated SH-SY5Y cells with or without NAC or MitoQ pretreatment. As shown in Fig. 2f–j, NAC or MitoQ pretreatment led to a significant reduction in MNNG-induced PAR formation, PARP-1 activation, and translocation of AIF into the nucleus. All these results indicated that the outburst of ROS (including mitochondria-derived ROS) contributes to parthanatos.

### ROS mediates parthanatos via ER calcium release and mitochondrial dysfunction

**Fig. 3:**
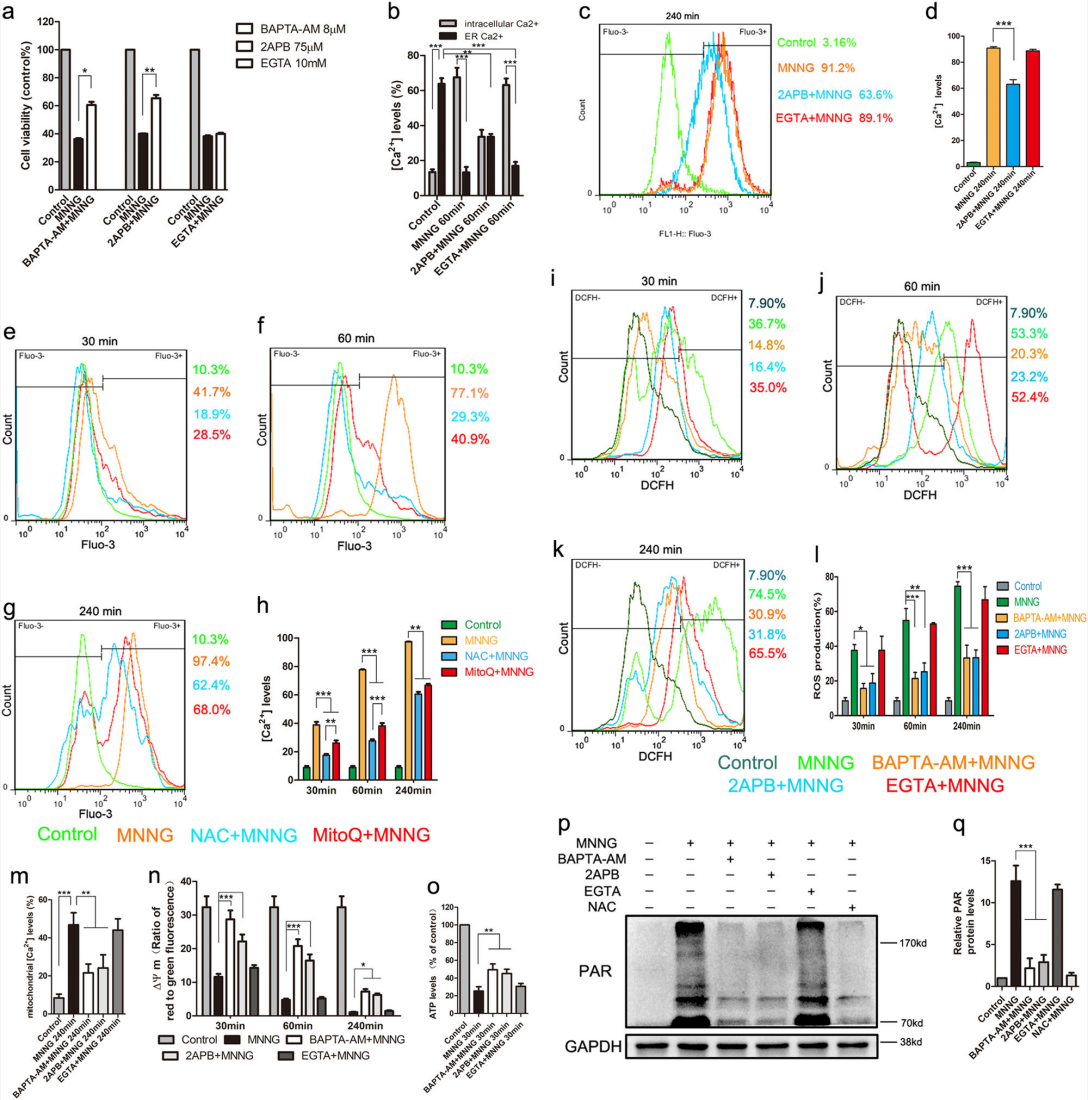
ER calcium release promotes mitochondrial depolarization in response to overproduction of ROS by MNNG-induced parthanatos. Cells were pre-treated with 8 μM BAPTA-AM, 75 μM 2APB, or 10 mM EGTA for 1 h, and then exposed to MNNG (250 μM) for 4 h. To measure ER calcium, we stimulated the cells with ionomycin (10 μmol/l). Cell viability was assessed by CCK8 assay (**a**), and Ca^2+^ levels were assessed by FACS analysis (**b**–**d**). **b**–**d** Quantification of Ca^2+^ levels in all groups. SH-SY5Y Cells were pre-treated with 5 mM NAC or 20 nM MitoQ for 1 h, followed by 250 μM MNNG for the indicated time. MNNG-induced Ca^2+^ levels were tested by FACS analysis at 30 min (e), 60 min (**f**), and 240 min (**g**). **h** The comparison of Ca^2+^ levels in all groups. SH-SY5Y Cells were pre-treated with 8 μM BAPTA-AM, 75 μM 2APB, or 10 mM EGTA for 1 h, followed by 250 μM MNNG for the indicated time. ROS production was tested by FACS analysis at 30 min (**i**), 60 min (**j**), and 240 min (**k**). **l** Quantification of ROS production in all groups. **m** FACS analysis at 60 min tested mitochondrial Ca^2+^ levels. **n** Flow cytometry assessed mitochondrial membrane potential with JC-1 staining. **o** ATP production was tested at 30 min. **p** Western blot analysis of PAR formation in different groups and GAPDH was used as a loading control. **q** Quantification of PAR expression. Each bar represents the mean ± S.E.M. from three independent experiments, *P < 0.05, **P < 0.01, ***P < 0.001

Intracellular Ca^2+^ is crucial to cell death pathway, and it also can be induced by excessive ROS generation^23^. To observe the change of intracellular calcium levels in MNNG (250 μM) treatment, we marked cytosolic calcium with fluo-3 AM. MNNG significantly increased the level of intracellular Ca^2+^ after 30 min of treatment and continued to increase up to 240 min (Fig. 3e–h). Similarly, in primary neurons, OGD/reoxygenation increased the level of intracellular Ca^2+^ (Figs. 2c, d). Meanwhile, MNNG-induced cell death and PAR generation could be partially reversed by intracellular free-calcium chelator, BAPTA-AM, (8 μM) pretreatment, which indicated that intracellular Ca^2+^ is critical for MNNG-induced parthanatos in SH-SY5Y cells (Fig. 3a, p, q).

To analyze the origin of Ca^2+^, we measured the calcium in the cytosol and ER. We found that MNNG caused a rapid increase of cytosolic calcium in parallel with a reduction of calcium in ER (Fig. 3b). To further analyze the origin of Ca^2+^, cells were pre-treated with 2APB (an IP3R inhibitor) or EGTA (an extracellular calcium chelating agent), which blocks ER calcium release and extracellular calcium entry, respectively. 2APB pretreatment not only reduced the basal levels of cytosolic Ca^2+^ and increased the levels of ER calcium (Fig. 3b–d), but also inhibited MNNG-induced cell death and PAR generation (Fig. 3a, p, q). On the other hand, extracellular calcium blocker, EGTA pretreatment not only failed to block MNNG-induced ER Ca^2+^ release to cytosolic, cell death, and PAR generation-rephrase (Fig. 3a–d, p, q). These results indicated ER-released Ca^2+^ contributes to MNNG-induced parthanatos, but not extracellular Ca^2+^.

Since both ROS and Ca^2+^ are critical to parthanatos, we wondered if a possible relationship between calcium and ROS production in parthanatos. Although both NAC and MitoQ pretreatment could significantly inhibit the calcium increase from 30 min up to 240 min (Fig. 3e–h), NAC has proven more effective in inhibiting the calcium increase than MitoQ within 30 min and 60 min, but not in 240 min (Fig. 3e–h). Similarly, in primary neurons, NAC and MitoQ could significantly inhibit intracellular Ca^2+^ (Figs. 2c, d). These results indicated that both intracellular ROS and mitochondria ROS could induce cytosolic calcium increase and the effect of intracellular ROS on cytosolic calcium is earlier than that of mitochondria ROS. Meanwhile, BAPTA-AM or 2APB pretreatment but not EGTA-decreased ROS production from 30 to 240 min, proving that ER calcium, but not extracellular calcium release is responsible for intracellular and mitochondria ROS (Fig. 3i–l).

Also, we also found MNNG treatment could cause the levels of mitochondrial Ca^2+^ to increase at 240 min, mitochondrial depolarization in a time-dependent manner and decreased ATP production-rephrase this entire sentence (Fig. 3m–o). Moreover, compared with MNNG-treated group, BAPTA-AM or 2APB pretreatment reduced the levels of mitochondrial Ca^2+^, increased the ATP production and maintained mitochondrial membrane potential at a higher level, whereas EGTA had no such effect (Fig. 3m–o). Taken together, these results demonstrated that intracellular ROS promotes ER calcium releasing, which leads to mitochondria calcium overload and mitochondria depolarization, and ultimately, caused the increase of mitochondria ROS.

### Propofol protects SH-SY5Y cells or primary neurons from parthanatos

**Fig. 4:**
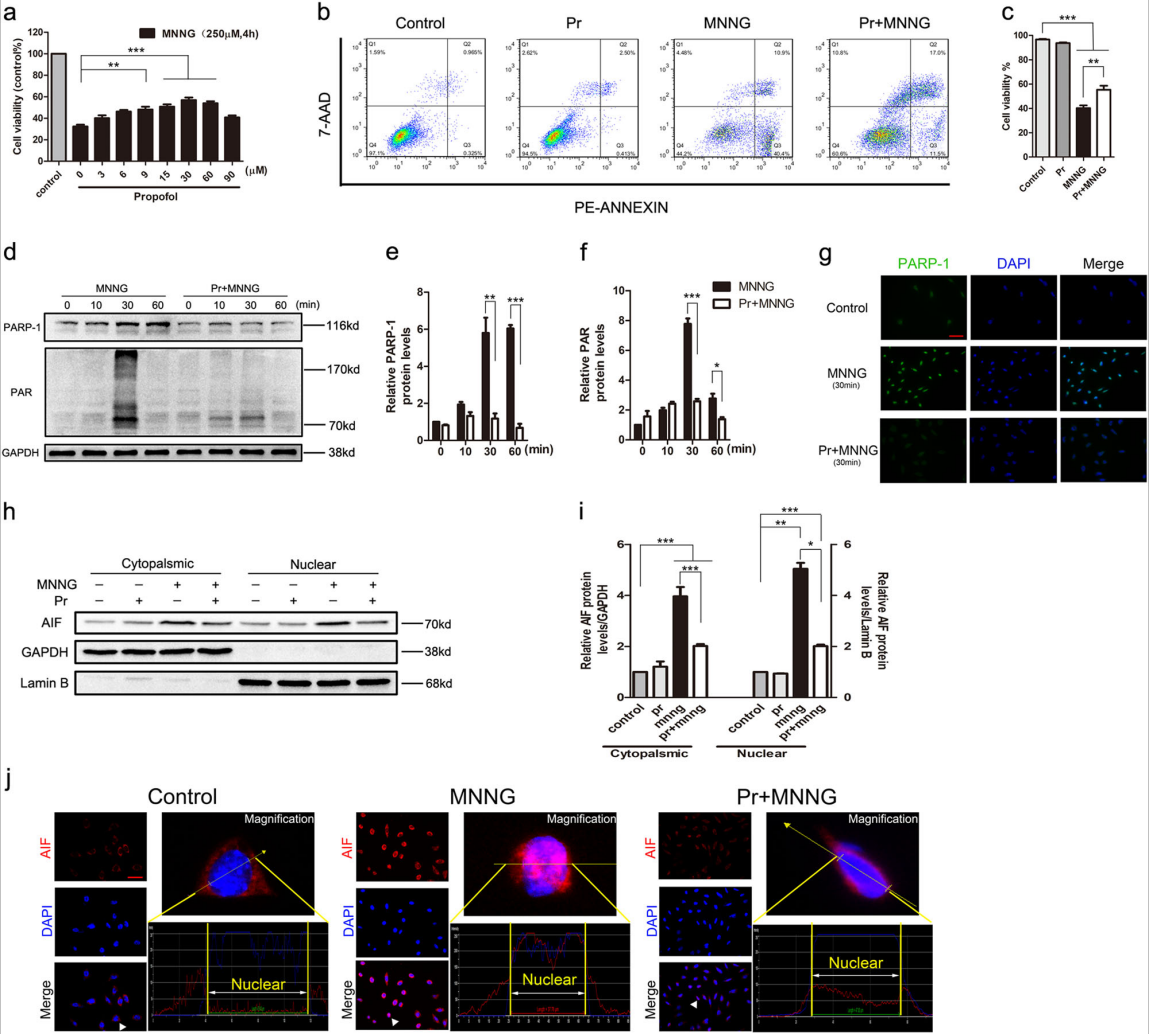
Propofol prevents MNNG-induced cell death via inhibited parthanatos. **SH-SY5Y cells were treated with MNNG (250 μM, 4 h) in the presence or absence of the indicated concentration of propofol for 1 h. a** CCK8 assay assessed cell viability. SH-SY5Y cells were exposed to MNNG (250 μM, 4 h) with or without 30 μM propofol pretreatment for 1 h. **b** Flow cytometry tested cell viability. **c** The comparison of cells viability in all groups. **d** Western blot analysis of PAR and PARP-1 in SH-SY5Y cells. The cells were pre-treated with 30 μM propofol for 1 h, then exposed to MNNG (250 μM) for the indicated time. GAPDH was used as a loading control. **e**, **f** Quantification of PARP-1 (**e**), and PAR (**f**) expressions. **g** Fluorescence microscopy analysis of PARP-1. **h** Western blots of subcellular fractions analysis translocation of AIF after MNNG (250 μM, 1.5 h) treatment in the presence or absence of propofol (30 μM, 1 h) in cells. **i** Quantification of AIF expression in both cytoplasm and nuclei. **j** Representative fluorescence images of AIF translocation. AIF (red) is seen in well-defined mitochondria-like structures in, and no AIF is seen in the nucleus (blue) in control, whereas a clear nuclear translocation and shrinkage of AIF (pink) is seen in MNNG treatment and inhibited by propofol. The graphs (down panel) show the fluorescence intensity profiles in two fluorescence channels along the arrow (up panel; Scale bar, 200μm.). These experiments have been replicated in three separate experiments. Each bar represents the mean ± S.E.M, *P < 0.05, **P < 0.01, ***P < 0.001

To determine whether propofol could protect SH-SY5Y cells or primary neurons from MNNG or OGD/reoxygenation-induced cell death, we treated SH-SY5Y cells with 250 μM MNNG, in combination with or without propofol treatment. Incubation with MNNG for 4 h caused cell death in more than 60% of SH-SY5Y cultures, but it was ameliorated in a dose-dependent manner by simultaneous incubation with propofol. Cells viability reached a peak at 30 μM propofol and gradually decreased until 90 μM, which was insignificant for MNNG-induced cell death (Fig. 4a). Meanwhile, flow cytometry analysis demonstrated as well that the living cells improved significantly when SH-SY5Y cells were treated with 30 μM propofol 1 h before exposure to MNNG (Fig. 4b, c). Similarly, primary neurons were treated by reoxygenation 24 h after OGD 12 h, in combination with or without 30 μM propofol treatment. As shown in Figs. 3a, OGD/reoxygenation-induced propofol inhibited cell death. The data indicated that propofol inhibits MNNG or OGD/reoxygenation-induced cell death.

Under these conditions, we next determined the effect of propofol on parthanatos. Western blotting analysis showed that pretreatment of 30 μM propofol suppressed PARP-1 expression and PAR formation for 30 min and 60 min (Fig. 4d–f). Similarly, propofol also suppressed PAR formation in primary neurons (Figs. 3b, c). To confirm the western blotting analysis, PARP-1 was performed after exposure to MNNG (250 μM, 30 min) followed by confocal microscopy, which obtained the similar result (Fig. 4g). Also, propofol prevented MNNG-induced AIF translocation as assessed by subcellular fractionation using immunoreactivity for Lamin B and GAPDH to monitor the purity of the nuclear and cytosolic fractions, respectively (Fig. 4h, i); and via confocal microscopy (Fig. 4j). Taken together, it appears that propofol protected MNNG or OGD/reoxygenation-induced cell death, which is associated with parthanatos, involving PARP-1 activation, PAR formation, and AIF translocation to nuclei.

### Propofol prevents parthanatos through inhibiting ROS overproduction and Ca^2+^ release

**Fig. 5:**
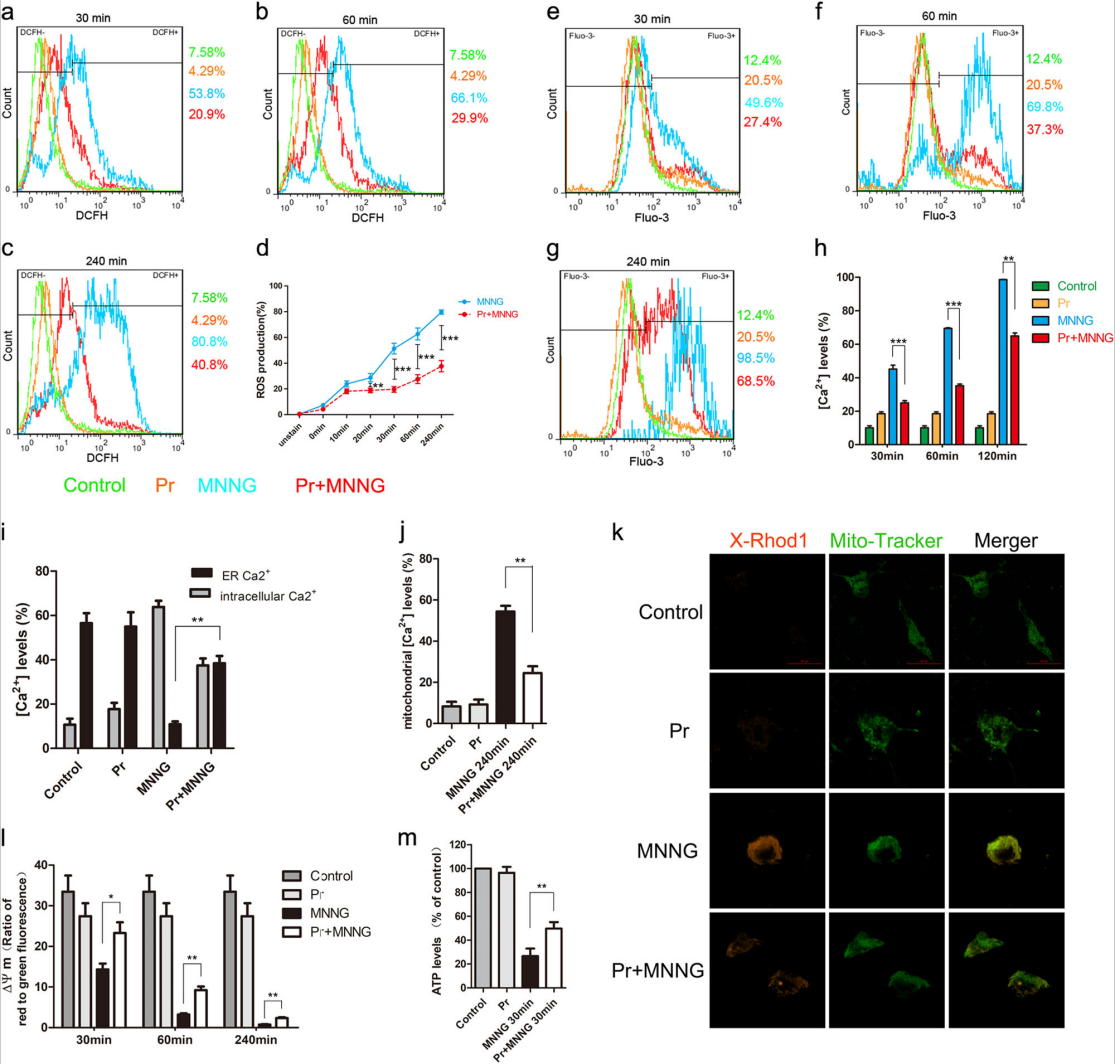
Propofol inhibits parthanatos via mitigated ROS production, Ca^2+^ releasing and mitochondrial depolarization. Cells were pre-treated with 30 μM propofol for 1 h, then exposed to MNNG (250 μM) at different time points. To measure ER calcium, we stimulated the cells with ionomycin (10 μmol/l). **a**–**c** ROS production was measured by FACS analysis with DCFH2-DA at 30 min (**a**), 60 min (**b**), and 240 min (**c**). **d** The comparison of ROS production at the indicated time. **e, f** Ca^2+^ was tested by FACS analysis at 30 min (**e**), 60 min (**f**), and 240 min (**g**). **h**, **i** The comparison of Ca^2+^ elevation in all groups. **j**, **k** Mitochondrial Ca^2+^ levels were tested by FACS analysis (**j**) and immunofluorescence analysis (**k**) at 60 min. **l** Flow cytometry measured mitochondrial membrane potential with JC-1 staining in all groups. **m** ATP production was tested at 30 min. These data were shown as mean ± S.E.M. from three independent experiments. *P < 0.05, **P < 0.01, ***P < 0.001

Experimental studies revealed that propofol could protect against cell death through antioxidant activity^15,16^. In our experiment, we have already confirmed that ROS contributes to MNNG or OGD/reoxygenation-induced parthanatos. We wondered whether ROS is involved in the mechanism underlying propofol inhibited parthanatos. To test this hypothesis, we monitored the intracellular ROS with or without propofol. In SH-SY5Y cells, we found propofol significantly reduced ROS production at 20 min, in a time-dependent manner, but within 10 min had no such effect (Fig. 5a–d). In primary neurons, propofol also reduced OGD/reoxygenation-induced ROS production (Figs. 3d, e). These results help to elucidate the mechanism of ROS-induced parthanatos, which was shown to be prevented by propofol.

We next investigated the effect of propofol on intracellular Ca^2+^. Intracellular Ca^2+^ significantly increased at 30 min after exposure to MNNG. As the incubation time extended to 60 and 240 min, the Ca^2+^ release was gradually elevated compared with those of the control and 30 min incubation groups (Fig. 5e–h). Propofol pretreatment significantly reduced MNNG-induced rise of intracellular Ca^2+^ at each incubation time point, whereas it increased the levels of ER Ca^2+^ (Fig. 5i). However, there was no significant difference in the effect of propofol treatment without the MNNG-induced rise of intracellular Ca^2+^ (Fig. 5e–i). These data indicated that propofol decreased the concentration of intracellular Ca^2+^, but increased the levels of ER Ca^2+^.

Also, under these conditions, we tested the relationship between propofol and mitochondria. As expected, propofol significantly reduced MNNG-induced rise of mitochondria Ca^2+^ and increased ATP production (Fig. 5j, k, m). Also, we found that administration of propofol inhibited mitochondrial depolarization at the indicated time caused by MNNG (Fig. 5l), which led to increasing ATP production (Fig. 5m). Taken together, these results suggested that the inhibitory effect of propofol on parthanatos is involving inhibition of ROS production, ER Ca^2+^ release, and mitochondrial depolarization.

### Propofol protects against I/R through ameliorating parthanatos

**Fig. 6:**
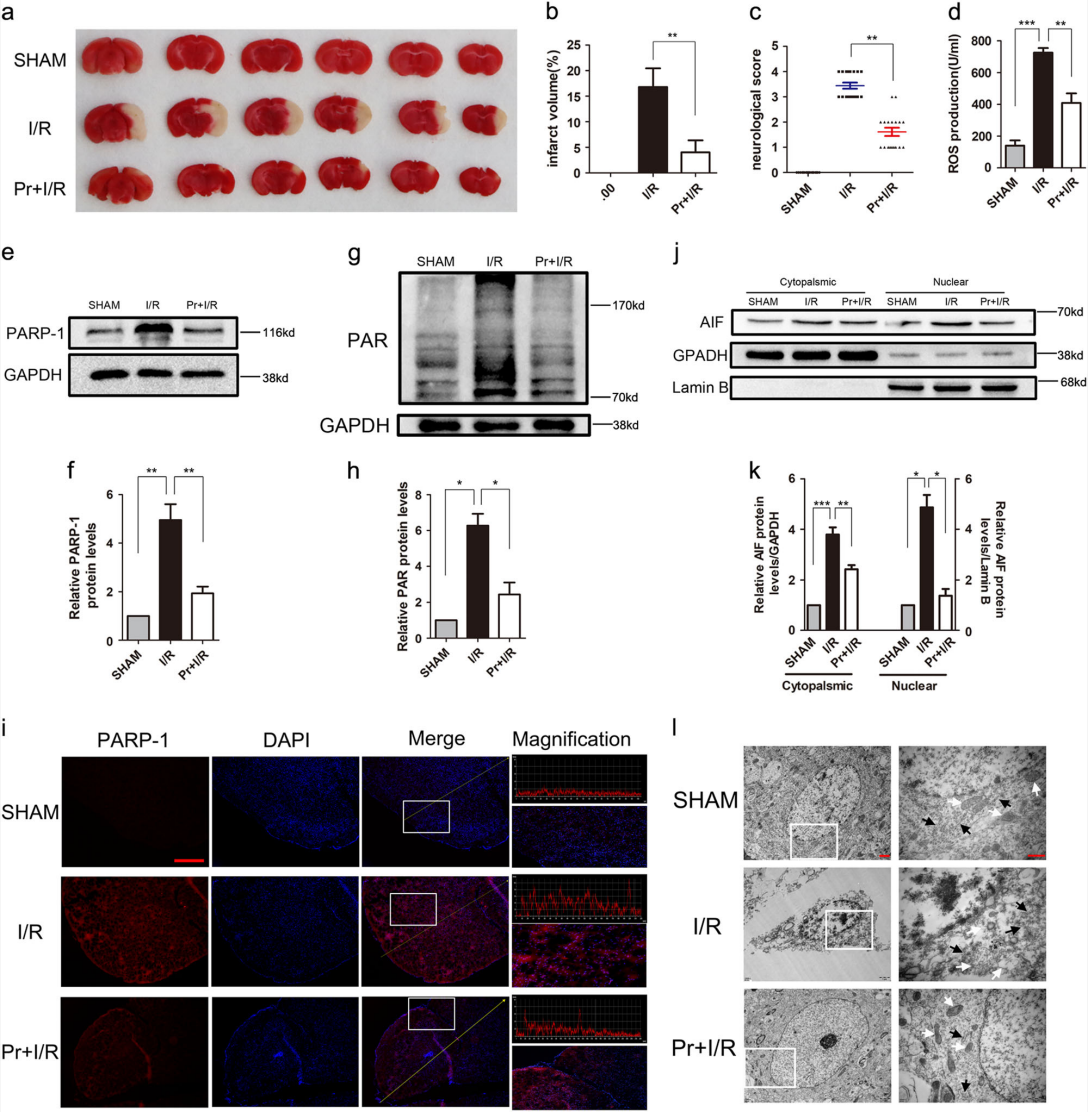
Mitochondria and ER are involved in propofol-attenuated cerebral I/R-induced parthanatos. C57BL/6 mice were injected with or without propofol (60 mg/kg) 24 h after 45 min of middle cerebral artery occlusion (MCAO). **a** Representative images of triphenyl tetrazolium chloride (TTC) staining of mice in all groups (*n* = 3 per group). **b** The quantification of infarction volume in all groups. **c** An open field evaluated neurological score on a scale of 0 to 5 (*n* = 18 per group). **d** The ROS production in all groups. Western blot analysis of parthanatos-related proteins, including PARP-1 (**e**), PAR (**g**), and AIF (**k**). Quantification of PARP-1 (**f**), PAR (**h**), and AIF (**k**) expressions. **i** Representative images of PARP-1 by fluorescence microscopy. Sections were co-stained with PARP-1 (red) and DAPI (blue). Higher magnification is shown (lower right). The graphs (upper right) show the fluorescence intensity profiles in the red fluorescence channel along the arrow (merger panel; scale bar, 500 px.). **l** Electron micrograph of mitochondria (white arrow) and ER (black arrow) in all groups. Lower magnification is shown (left, Scale bar, 1 μm); Higher magnification is shown (right; scale bar, 500 nm). These experiments have been replicated in three separate experiments. Each bar represents the mean ± S.E.M, *P < 0.05, **P < 0.01, ***P < 0.001

Much experimental evidence indicated that propofol had a neuroprotective effect against cerebral I/R injury^19,24^. Besides, many studies have shown that parthanatos has a critical role in lots of neurologic diseases including cerebral I/R. We next focused on the effect of propofol on parthanatos in cerebral I/R in vivo. We measured infarct size and neurological scores in the reperfusion brain 24 h after MCAO with or without propofol (60 mg/kg) in vivo. Mice were challenged with MCAO for 60 min and examined with 2,3,5-triphenyl-2H-tetrazolium chloride (TTC) staining after 24 h of reperfusion. We found treatment with propofol led to ~20% reduction in infarct volume, compared with those in WT mice, after reperfusion 24 h (Fig. 6a, b). Also, the neurological score was improved by 4 points in mice of treatment with propofol (Fig. 6c). Therefore, results showed that propofol has a critical (redundant) effect in neuroprotection against brain I/R.

Based on the neuroprotection of propofol, we proposed that the brain protective mechanism of propofol is through inhibiting parthanatos. Therefore, to test this hypothesis, we investigated the ROS production and parthanatos-related proteins in propofol treatment in vivo. Propofol could decrease the I/R-induced rise of ROS production in plasma (Fig. 6d). Western blotting analysis showed that sham controls had low PAR formation and PARP-1 levels, whereas cerebral I/R-induced higher levels of PAR formation and PARP-1 overproduction than sham group. Propofol treatment completely blocked cerebral I/R-induced PAR formation and PARP-1 overproduction at 24 h after reperfusion (Fig. 6e–h). To confirm the western blotting analysis, PARP-1 was detected after reperfusion at 24 h followed by brain tissue confocal microscopy, which obtained similar results (Fig. 6i). Also, subcellular fraction results indicated that propofol could inhibit AIF translocation into the nucleus in cerebral I/R injury (Fig. 6j, k). To determine whether suppression of mice cerebral I/R-induced parthanatos may be due to the protection of mitochondria and ER by propofol, we examined ultrastructural changes in the mitochondria and ER after reperfusion at 24 h by transmission electron microscopy. Neurons in the sham appeared to be standard structures, with relatively healthy-looking ER, mitochondria, and nuclei. However, abnormal mitochondria and ER were in the majority of the cerebral I/R group, with swollen, vacuolated mitochondria plus cristae remodeling and dispersed and swollen ER. Propofol ameliorated the damage, with slight swelling in mitochondria plus less vacuolar degeneration and fragments of cristae and more contiguous and less swollen ER than the cerebral I/R group (Fig. 6l). These results suggested propofol suppressed parthanatos through mitochondrial damage and ER during cerebral I/R.

## Discussion

**Fig. 7:**
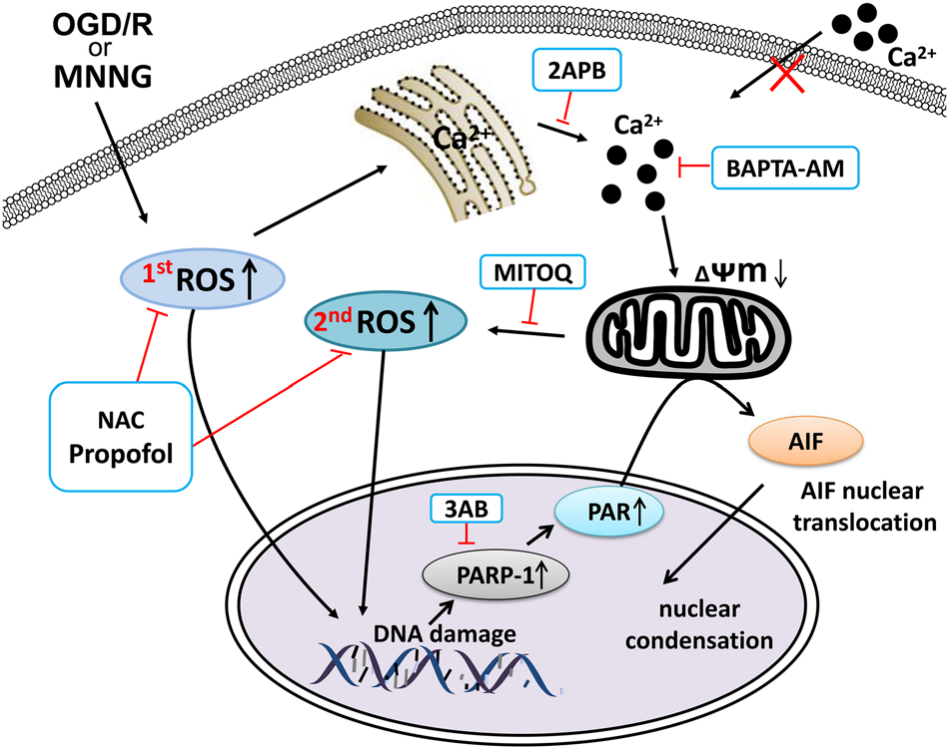
The ‘ROS–ER–Ca^2+^–mitochondria’ loop in parthanatos and the role of propofol in parthanatos. MNNG or OGD/reoxygenation induces the generation of ROS, which can directly lead to parthanatos or target ER calcium channels (IP3R), leading to the release of calcium from the ER into the cytosol. As a result, mitochondria take up Ca^2+^, which leads to mitochondrial depolarization, and in turn promotes mitochondrial ROS production. Mitochondrial ROS can lead to parthanatos or further increase Ca^2+^ release from ER, resulting in a vicious feedback cycle. Besides, Propofol can inhibit the generation of ROS and Ca^2+^, which in turn inhibits parthanatos

Massive DNA damage leads to PARP-1 hyperactivation, which causes intracellular NAD+ depletion, and inhibition of ATP production. Moreover, NAD+ can be transformed into PAR polymers by overactivation of PARP-1, and then PAR triggers the translocation of AIF from mitochondria to the nucleus, where it causes chromatin condensation, DNA fragmentation, and finally cell death (parthanatos)^2,25^. Such DNA damage occurs by oxidation and an increase in cytosolic calcium^13,26^. Recent studies show that ROS and calcium have been well established in parthanatos^12,27^. But the detailed mechanisms are unclear. In this study, we identify the regulating mechanisms of ROS and calcium in mediating parthanatos through the ROS–ER–calcium–mitochondria signal pathway (Fig. 7).

An imbalance of intracellular calcium concentration is a cell death signal, and ROS can trigger the increased intracellular calcium. Accumulating evidence suggests that ROS contributes to DNA damage and PARP-1 hyperactivation, the excess of which, can result in PARP-1-dependent cell death (parthanatos)^11,12^. Some studies have demonstrated that calcium was required for the PARP-1 hyperactivation in the model of oxidative stress-induced cell death^28,29^. Consistently, in our results, the production of ROS and calcium are increased in MNNG-induced parthanatos. When NAC or BAPTA-AM pretreatment directly blocked increases in ROS or calcium, parthanatos was inhibited. We first found that decreasing the production of ROS can inhibit the increase of calcium. Notably, accumulating evidence suggests that ROS has a critical role in calcium balance. Zhang et al.^29^ reported that intracellular calcium is required for ROS-induced PARP-1 hyperactivation in non-apoptotic cell death. Furthermore, Robert et al.^30^ found that mitochondrial ROS is required for elevation of cytosolic calcium. Interestingly, in this study, when we blocked the mitochondrial ROS by MitoQ, parthanatos was suppressed, and intracellular calcium concentration reduced. Besides, the effect of NAC on inhibiting parthanatos and calcium concentration was more effective than MitoQ. Moreover, when we inhibited NADPH oxidases by apocynin, cell death and ROS overproduction were suppressed. Therefore, our study demonstrated that not only cytoplasmic ROS but also mitochondrial ROS induced increasing calcium in parthanatos.

Our results have proved that increasing calcium, which can be induced by ROS, is involved in parthanatos. However, the origin of calcium is unclear. As we all know, intracellular calcium concentration must be precisely regulated, and this delicate balance is maintained by extracellular membrane calcium channels, intracellular calcium release channels, and calcium pumps, and exchanges^26,31,32^. The endoplasmic reticulum (ER), a specialized organelle, is involved in intracellular calcium storage and released^33^. Previous studies have identified that ROS can increase calcium release from the ER by stimulating ER–calcium release channels, such as an inositol-1,4,5-trisphosphate receptor (IP3R)^34–36^. IP3R-mediated ER Ca^2+^ mobilization is activated by ROS, which is induced by NADPH and mitochondrial enzymes. Besides, the increasing intracellular calcium concentration is also dependent on Ca^2+^ store depletion mediated by Ca^2+^ entry, termed store-operated Ca^2+^ entry (SOCE)^37^. The channel responsible for mediating Ca^2+^ entry secondary to ER-stored Ca^2+^ depletion is termed store-operated Ca^2+^ entry channels (SOCs), such as STIM and Orai CRAC channels^37,38^. Previous reports have found that ROS generation can activate SOC entry^39,40^. Currently, there is little understanding regarding the origin of calcium in parthanatos, although Janssen et al.^41^ reported that ER calcium could activate PARP-1 in cell death. Therefore, in this study, we found that calcium contributed to parthanatos from ER through IP3R. Although pretreatment with 2APB (also a putative SOCE inhibitor) can inhibit parthanatos, we also found that EGTA (an extracellular calcium chelator) could not inhibit parthanatos, which means calcium from ER is releasing but does not trigger SOCE in parthanatos. On the contrary, Frances et al.^42^ found that ROS-induced store-operated Ca^2+^ entry coupled with PARP-1 hyperactivation accumulates PAR polymers in a non-apoptotic cell death pathway. This type of cell death is via an AIF-independent pathway^29^, which is different from parthanatos (AIF-dependent). Therefore, we thought that calcium from the ER does not trigger SOCE in parthanatos. ROS cause ER stress, which leads to activation of the downstream effector, CHOP, which then triggers Ca^2+^ release from ER^43^. The mechanism of ROS triggered ER stress may be related to the PERK–Eif2α–ATF4–CHOP pathway^44^, which we intend to explore in future studies. The calcium released from ER enters into the mitochondrial and causes mitochondrial depolarization, and increasing mitochondrial ROS production^45,46^. In our study, we found that calcium from ER entered the mitochondrial. When there is an overload of mitochondrial calcium, it leads to the loss of mitochondrial membrane potential and mitochondrial ROS overproduction, which, in turn, triggers cell death. Our data explain the loss of mitochondrial membrane potential and mitochondrial ROS overproduction, which contribute to parthanatos.

Propofol has previously been reported to have a positive effect on both antioxidation^16^ and neuroprotection^24^. Besides, some studies reported that parthanatos has a role in cerebral injury^6–9^, unusually cerebral I/R injury^2^. Accumulating evidence suggests that propofol inhibits apoptosis and autophagy via inhibiting ROS production both in vivo and in vitro^16,17^. Additionally, we have been shown that ROS is required for parthanatos. However, the mechanism of propofol-induced amelioration of parthanatos is not fully understood. Consistently, our results indicated that appropriate concentrations of propofol (30 μM) prevented parthanatos by inhibiting ROS overproduction in vitro. However, the effect gradually decreased with increasing concentrations up to 90 μM, because a high dose of propofol tends to decrease the cell survival^47^. It may indicate that the therapeutic effect of propofol is highly dose-dependent. Furthermore, propofol (60 mg/kg) protected cerebral I/R injury through inhibiting parthanatos. Also, previous studies have shown that propofol protected cerebral I/R injury via mitigation of calcium concentration and reduced Ca^2+^-induced mitochondrial dysfunction^20,48^. Conversely, some researchers have shown that propofol protected cerebral I/R injury, not through mitochondrial membrane potential^19^. However, it is widely believed that calcium and mitochondrial have a role in the neuroprotective effect of propofol. Also, mitochondrial dysfunction has also had a role in PARP-1-mediated cell death^1,49^. Our results suggest that propofol prevents parthanatos via reducing the level of intracellular calcium and mitochondrial dysfunction, via maintaining a mitochondrial membrane potential.

Moreover, propofol reduces mitochondrial swelling and leads to less swollen ER in cerebral I/R injury. The ROS that is synthesized, in excess, during and after ischemia may also cause mitochondrial dysfunction^50^, therefore, the inhibition of ROS production may prevent mitochondrial and ER swelling. Whether in vivo or in vitro, it is widely believed that propofol inhibits parthanatos through ROS-induced calcium releasing reduction, which in turn, attenuates mitochondrial dysfunction.

In conclusion, we have identified the regulating mechanisms of ROS and calcium in mediating MNNG or OGD/reoxygenation-induced parthanatos through the ROS–ER–calcium–mitochondrial signal pathway. Our results indicate that propofol inhibits the development of parthanatos in vivo and in vitro via reducing ROS production, Ca^2+^ releasing and mitochondrial depolarization.

## Materials and methods

### Cell culture and mice

SH-SY5Y cells were cultured in Dulbecco’s modified Eagle’s medium (DMEM, Gibco BRL) supplemented with 10% fetal bovine serum (FBS, Gibco BRL), 100 U/ml penicillin, and 100 µg/ml streptomycin at 37 °C in 5% CO_2_.

Primary cortical neurons were isolated from 14-days-old C57BL/6 mice. Detailed methods were provided in Supplementary Materials and Methods.

### CCK8 assay

Cell viability was assessed by Cell Counting Kit-8 (CCK8) assay following the manufacturer’s instructions (Dojindo, Japan).

### Western blotting

The expression of PARP-1, PAR, AIF, GAPDH, and Lamin B were analyzed by western blots as described in Supplementary Materials and Methods.

### ROS measurement

Intracellular ROS was measured by flow cytometry using DCFH_2_-DA (Beyotime Biotechnology, China) (see Supplementary Materials and Methods).

The concentration of ROS in mouse plasma was measured by mouse ROS ELISA kit (Albion, China) following the manufacturer’s instructions.

### Mn-SOD and NADPH activity detection

Mn-SOD activity was measured using the MnSOD Assay Kit with WST-8 (Beyotime Biotechnology, China) following the manufacturer’s instructions. NADPH activity was measured by NADPH ELISA kit (Albion, China) following the manufacturer’s instructions.

### Measurement of calcium level

Intracellular calcium, ER calcium, and mitochondrial calcium were tested by flow cytometry, and mitochondrial calcium was also measured by immunofluorescence as described in the Supplementary Materials and Methods.

### Measurement of ATP levels

Intracellular ATP content was analyzed with the EnzyLight™ ATP Assay Kit (BioAssay Systems, USA) following the manufacturer’s instructions (see Supplementary Materials and Methods).

### Cellular NAD^+^ determination

SH-SY5Y cells were plated in 10 cm^2^ plates at a density of 1 × 10^7^ cells per well. After the various treatments, cells were collected and resuspended in 100 μl NAD^+^ extraction buffer. Extracts were heated (5 min) at 60 °C, and 20 μl of assay buffer was added followed by the extraction buffer. Mixtures were vortexed and centrifuged at 13,000 rpm for 5 min. Supernatants were added to the working reagent containing assay buffer (Bioassay Systems, USA). Optical density at 565 nm was recorded at time zero and 15 min using a 96-well plate reader spectrophotometer.

### Mitochondrial membrane potential (JC-1) assay

Mitochondrial membrane potential was measured by flow cytometry following the manufacturer’s instructions (see Supplementary Materials and Methods).

### FACS analysis cell death

SH-SY5Y cell death was detected by staining with PE Annexin V Apoptosis Detection kit (BD Biosciences) (see Supplementary Materials and Methods).

### Immunofluorescence analysis

PARP-1 expression and AIF localization were analyzed by immunofluorescence analysis as described in Supplementary Materials and Methods.

### Middle cerebral artery occlusion (MCAO)

Cerebral ischemia–reperfusion was induced by MCAO as previously described^51,52^. Detailed methods were provided in Supplementary Materials and Methods.

### Transmission electron microscopy

The tissues were fixed for 1 h with 2.5% glutaraldehyde. After dehydration, tissues were embedded into epon; the ultra-thin section was obtained using microtome and sections were placed on copper electron microscopy grids. Sections were stained with uranyl acetate (5 min), and then lead citrate (2 min). Micrographs were observed in transmission electron microscope (Hitachi H7500 TEM, Tokyo, Japan).

### Statistical analysis

Data were analyzed in SPSS19.0 (California, USA). Two groups were determined with Student’s *t*-test (two-tailed), whereas analysis of variance (ANOVA) assessed comparisons among multiple groups. All graphs represent the mean ± S.E.M. *P* values of <0.05 were considered significant. To ensure adequate power to detect the effect, we performed at least three independent tests for all molecular biochemistry studies and at least five mice from three different litters for animal studies

## Electronic supplementary material

Supporting Information

Figs. 1

Figs. 2

Figs. 3
