# Supplementary material for: Propofol inhibits parthanatos via ROS–ER–calcium–mitochondria signal pathway in vivo and vitro
Source: Cell Death Dis. 2018 Sep 17;9(10):932. doi: 10.1038/s41419-018-0996-9 (PMC6141459; doi:10.1038/s41419-018-0996-9)
Supplement: Supplementary file 1 — Supporting Information [file 41419_2018_996_MOESM1_ESM.docx]

Supporting Information

**Materials and Methods**

**Primary cortical neurons culture and mice**

Primary cortical neurons were isolated from 14-days-old C57BL/6 mice. The fetal mouse was rapidly decapitated before cortices were removed. The dissociated cerebral cortices were then digested with 0.2% trypsin at 37°C and terminated with trypsin inhibitor (Sigma, USA) after 2 minutes. The dispersed cells were collected and plated on poly-D-lysine pre-coated 6-well plates in neurobasal medium (Gibco) supplemented with 2% B27, 2 mM glutamine, 100 units/ml penicillin and 100 µg/ml streptomycin. 2 days later, 10 µM cytosine β-D-arabinofuranoside (Sigma) was added, followed by changing the culture medium after 24 hours.

C57BL/6 mice (14-days-old or 8-weeks-old, 26-28g) were purchased from the Central Animal Facility of Southern Medical University. All experimental animal protocols conformed to National Institutes of Health guidelines and approved by the committees of Southern Medical University (Guangzhou, China, L2015046).

**Reagents and antibodies**

Propofol (56931), NAC (A7250), 2APB (D9754) and EGTA (E0396) were obtained from Sigma. BAPTA-AM (S7534), Z-VAD-FMK (S7023) and 3AB (S1132) were obtained from Selleck.cn. MNNG was obtained from TCI ([M0527)](http://www.tcichemicals.com/eshop/zh/cn/commodity/M0527/). MitoQ was from Focus Biomolecules (10-1363). Antibodies specific for PARP-1 (9532), AIF (5318), GAPDH (2118), Lamin B (12586), horse anti-mouse IgG (H&L) (7076) and goat antirabbit IgG (H&L) (7074) were obtained from Cell Signaling Technology. PAR antibody (AM80) was from Merck Millipore. Alexa Fluor 488 goat anti-rabbit (A-11034) and Alexa Fluor 555 goat anti-rabbit (A32732) secondary antibodies were obtained from Thermo Fisher Scientific.

**CCK-8 assay**

Cell viability was assessed by Cell Counting Kit-8 (CCK8) assay following the manufacturer’s instructions (Dojindo, Japan). In brief, SH-SY5Y cells were seeded in 96-well plates with 1×10^5^/well of cell density and cultured for 24 hours. Cells were pretreated with indicated concentrations of indicated reagents for 1 hour and then treated with indicated concentrations of MNNG for 4 hours. 10μL CCK-8 was added, after incubating for 2 h, and absorbance was detected at 450 nm using a Microplate Reader (Thermo Scientific, China).

**Western blotting**

Cells or tissue lysates were obtained using RIPA Lysis (Beyotime Biotechnology, China). The lysates were centrifuged, and the supernatants were determined by BCA kit (ThermoFisher Scientific). 50μg proteins were separated by 8 and 12% SDS-PAGE and then were transferred to PVDF membranes (Millipore). After blocking with 5% skimmed milk for 1 h at room temperature, the membrane was incubated with primary antibodies to PARP-1 (1:1000), PAR (1:500), AIF (1:1000), GAPDH (1:1000) and Lamin B (1:800) at 4℃ overnight. Then the membrane was incubated with secondary antibodies for 1 h at room temperature, followed by visualization using ECL reagent (Millipore) and the protein bands were obtained using a chemiluminescence detection system (Image Station 4000R, KODAK, USA). The gray value of protein was measured Image Pro Plus.

**Reactive Oxygen Species measurement**

SH-SY5Y cells at a density of 2*105 cells/well in a 6-well plate were cultured and pretreated with indicated reagents. At indicated time points following exposure to MNNG (250μM), at the end of treatment, cells were loaded with fluorescent dye DCFH_2_-DA (Beyotime Biotechnology, China) in serum-free DMEM and incubated for 30 min at 37℃ in the dark. Cells were then washed twice with PBS after suspended in fresh DMEM; the fluorescence intensity was measured by FACScan flow cytometry (BD Biosciences) at Ex./Em.-488/525 nm. Data were analyzed using FlowJo (TreeStar, USA) The resultsexpressed as a percentage of the favorable rate.

The concentration of ROS in mouse plasma was measured by mouse ROS ELISA kit (Albion, China) following the manufacturer’s instructions.

**Measurement of calcium level**

SH-SY5Y cells were plated in 6-well plates at a density of 2*105 cells/well. Cells were pre-treated with different reagents for 1 h and then incubated with MNNG (250μM) for indicated time points. To measure ER calcium, we stimulated the cells with ionomycin (10μmol/L). At the end of treatment, cells were loaded with 10μM Fluo-3 AM (Beyotime Biotechnology, China), which test intracellular calcium or 5μM Rhod-2 AM (Santa Cruz, USA), which test mitochondrial calcium, for 30 minutes in the dark. After being washed with PBS twice, cells were resuspended in 500ml PBS and immediately analyzed by flow cytometry at an Ex./Em.-488/525 nm or Ex./Em.-549/578. Data were analyzed using FlowJo (TreeStar, USA). The resultsexpressed as a percentage of the favorable rate.

Cells were seeded in glass slides. After necessary treatment, cells were loaded with fluorescent dye 5μM X-Rhod1 (Santa Cruz, USA) in PBS and incubated for 30 min at 37℃ in the dark. Moreover, then loaded with 100nM Mito-Tracker Green (Beyotime Biotechnology, China) for 30 min at 37℃ in the dark. Cells were then washed twice with PBS, and images were taken on a confocal microscope (Nikon Corporation).

**Measurement of ATP levels**

SH-SY5Y cells were plated in 96-well plates at a density of 1*10^4^ cells/well. Cells were treated with indicated reagents. Cells were lysed, and the total intracellular ATP content was analyzed with the EnzyLight™ ATP Assay Kit (BioAssay Systems, USA) following the manufacturer’s instructions.

**Mitochondrial membrane potential (JC-1) assay**

SH-SY5Y cells were seeded in 6-well plates at a density of 2*10^5^ cells/well. After the various treatments, the cells were collected and loaded with JC-1 for 30 min at 37℃ in the dark and then washed twice with PBS. The cells were resuspended in 500ml PBS and analyzed by flow cytometry at an Ex./Em.-530/590 (green/red) nm and data analyzed by the software FlowJo.

**FACS analysis cell death**

SH-SY5Y cell death was detected by staining with PE Annexin V Apoptosis Detection kit (BD Biosciences). After the various treatments, cells were collected, washed by Annexin V buffer and resuspended in 300 μl of binding buffer. The cells were stained with 3 μl PE Annexin and 3 μl 7-AAD for 15 min in the dark and then evaluated using flow cytometry, and the FlowJo analyzed the samples.

**Immunofluorescence analysis.**

Cells were seeded in glass slides, and mice brain was sliced into frozen sections. After necessary treatment, cells or sections were fixed with 4% paraformaldehyde (10 min at room temperature), permeabilized with 0.5% Triton X-100 in PBS (10 min) and block with 5% bovine serum albumin (BSA) for 2 h. Then cells or sections were incubated with the anti-PARP-1 antibody (1:200) or anti-AIF antibody (1:200) at 4℃ overnight, followed by secondary Alexa Fluor 488 goat anti-rabbit antibody (1:400) or Alexa Fluor 555 goat anti-rabbit antibody (1:400) for 1 h at room temperature. Nuclei were counterstained with DAPI (10 min) before images were taken on a fluorescence microscope (Nikon Corporation).

**Middle cerebral artery occlusion (MCAO)**

MCAO in this study induced focal cerebral ischemia. Mice were anesthetized with pentobarbital sodium and maintained rectal temperature at 36±0.5 ℃ by a homeothermic blanket. To establish MCAO, we inserted a 6-0 silicon-coated monofilament suture (Doccol, Redland, CA, USA) into the left external carotid artery, advanced into the internal carotid artery and occlude the origin of the MCA. After 45 min MCAO, the suture was removed for reperfusion. No operative death in each group (n=18), therefore in each group there were 18 mice. Sham-operated animals underwent internal carotid artery exposure without MCAO. Propofol (1mg/kg/h) injected by continuous enterocoelia pumping at the pre-operative (30 min), during the MCAO (45 min) and in the initial 30 min of reperfusion.

After reperfusion, the mice were euthanized, and their brains were immediately isolated. Mouse brains were cut into 1-mm coronal sections, and a total of six sections were collected. The sections were exposed to triphenyl tetrazolium chloride (TTC) staining solution for 20 min at 37℃ before fixed in 4% formalin solution overnight. The sections were imaged, and Image Pro Plus tested the infarct volume.

Neurological scores were assessed after reperfusion (24 h) using the following scale: 0, no neurological deficits; 1, failure to fully extend the right forepaw; 2, circling to the right; 3, falling to the right; 4, did not walk spontaneously and had depressed of consciousness.

**Figure legends**

**Figs. 1. OGD/Reoxygenation induces parthanatos in primary mouse neurons.** (a) Primary neurons were Reoxygenation 24h after OGD 6h or 12h with or without 10μM z-vad-fmk, 10μM chloroquine, 30μM 3-AB pretreatment for 1 h. The CCK8 assay assessed cell viability. (b) OGD12h/Reoxygenation24h-induced PAR accumulation. GAPDH was used as a loading control. (c) Quantification of PAR expressions. Each bar represents the mean±S.E.M based on three independent experiments. **P<0.01, ***P<0.001.

**Figs. 2. ROS and Ca2+ contribute to OGD/Reoxygenation induced parthanatos.** Primary neurons were Reoxygenation 24h after OGD 12h with or without 5 mM NAC, 20 nm MitoQ pretreatment for 1 h. (a) FACS analysis of DCFH2-DA fluorescence assessed ROS production. (b) Quantification of ROS production in all groups. (c) FACS analysis tested ca2+ levels. (d) The comparison of Ca2+ levels in all groups. Each bar represents the mean±S.E.M based on three independent experiments. **P<0.01, ***P<0.001.

**Figs. 3. Propofol inhibits OGD/Reoxygenation-induced parthanatos.** Primary neurons were Reoxygenation 24h after OGD 12h with or without 30μM propofol pretreatment for 1h. (a) The CCK8 assay assessed cell viability. (b) Western blot analysis of PAR accumulation. GAPDH was used as a loading control. (c) Quantification of PAR expressions. (d) FACS analysis measured ROS production. (e) Quantification of ROS production in all groups. Each bar represents the mean±S.E.M based on three independent experiments. **P<0.01, ***P<0.001.
