# Supplementary figures and images for: Propofol inhibits parthanatos via ROS–ER–calcium–mitochondria signal pathway in vivo and vitro
Source: Cell Death Dis. 2018 Sep 17;9(10):932. doi: 10.1038/s41419-018-0996-9 (PMC6141459; doi:10.1038/s41419-018-0996-9)

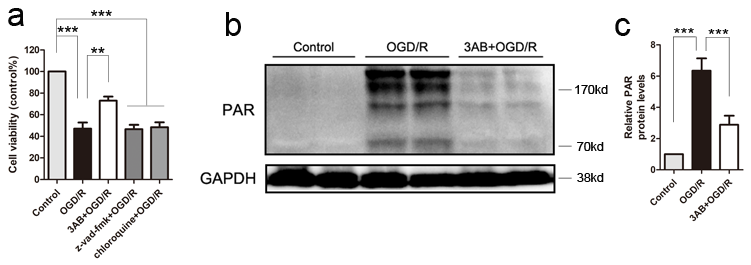

Supplement: Supplementary file 2 — Figs. 1 [file 41419_2018_996_MOESM2_ESM.tif]

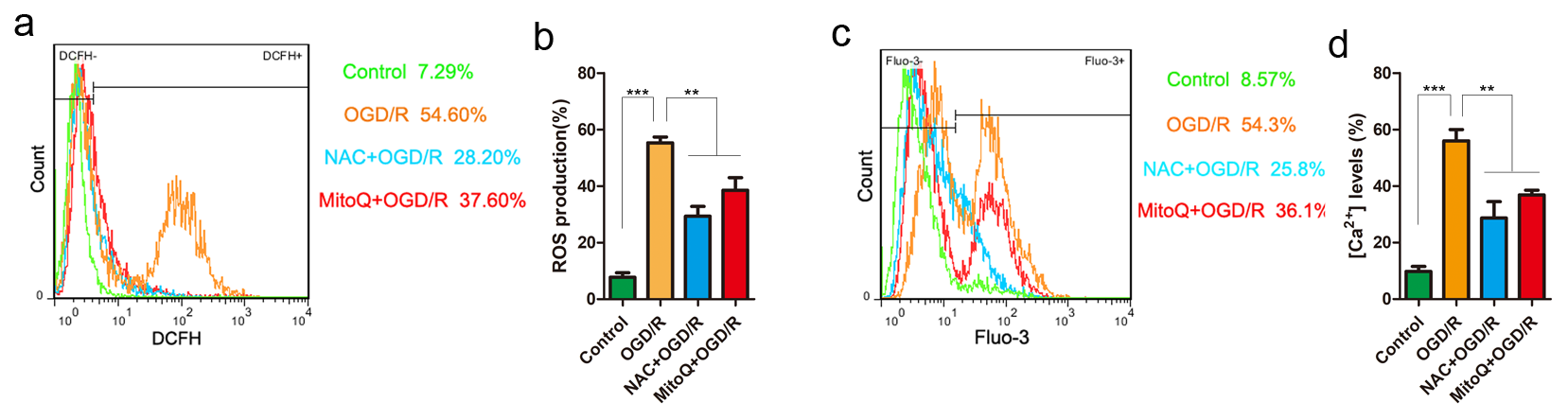

Supplement: Supplementary file 3 — Figs. 2 [file 41419_2018_996_MOESM3_ESM.tif]

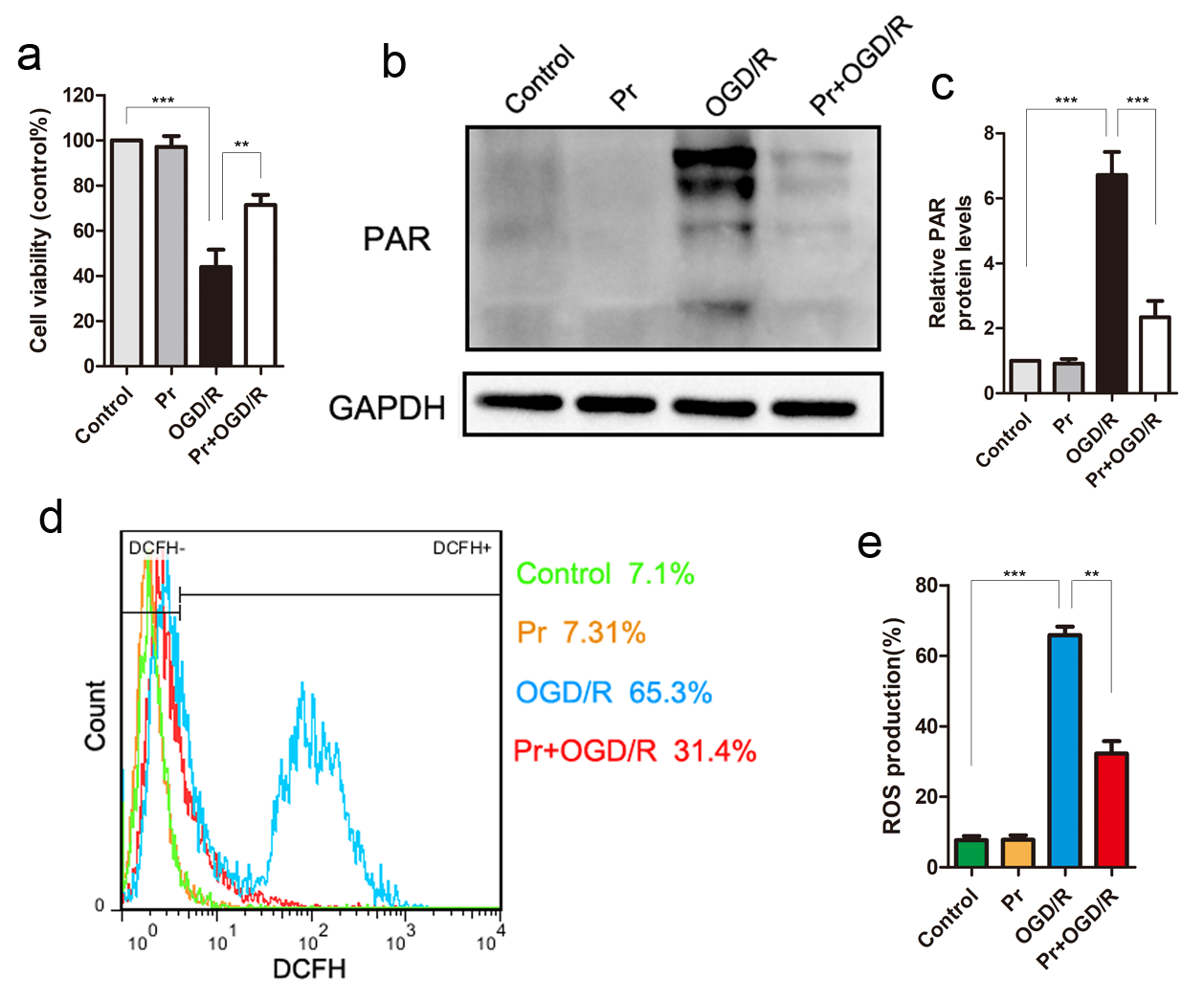

Supplement: Supplementary file 4 — Figs. 3 [file 41419_2018_996_MOESM4_ESM.tif]
